# PD‐L1 Promotes Immunological Tolerance and Enhances Visual Protection of hESC‐RPE Grafts in Retinal Degeneration

**DOI:** 10.1111/cpr.70007

**Published:** 2025-02-14

**Authors:** Bowen Li, Xue Zhang, Yajie Fang, Min Chen, Qiyou Li, Yuxiao Zeng, Chunge Ren, Chengang Wang, Yingxue Lv, Jia Lu, Hongling Liu, Yong Liu

**Affiliations:** ^1^ Southwest Hospital/Southwest Eye Hospital Third Military Medical University (Army Medical University) Chongqing China; ^2^ Key Lab of Visual Damage and Regeneration & Restoration of Chongqing Chongqing China; ^3^ Jinfeng Laboratory Chongqing China

**Keywords:** AMD, hESC‐RPE transplantation, immune checkpoints, immune tolerance, PD‐L1

## Abstract

Immune rejection is a major barrier to the successful human embryonic stem cell‐derived retinal pigment epithelial (hESC‐RPE) transplantation for age‐related macular degeneration (AMD). Traditional strategies to mitigate immune rejection involve ablating major histocompatibility complex (MHC) molecules on hESC‐RPE. An alternative approach is immune checkpoint overexpression, avoiding natural killer (NK) cell‐mediated destruction due to MHC‐I deficiency. Our study highlights the benefits of PD‐L1 overexpression without requiring MHC gene deletion, which preserved the immunosuppressive functions of hESC‐RPE on NK cells. In Vivo experiments in retinal degeneration models showed that PD‐L1‐expressing hESC‐RPE grafts exhibited significantly higher survival, reduced apoptosis and enhanced visual protection. Single‐cell transcriptomics revealed reduced immune activation and oxidative stress in PD‐L1‐overexpressing grafts. PD‐L1's protective role was further evidenced by improved light transduction in host photoreceptors. These findings support PD‐L1 overexpression as a promising strategy to improve the efficiency of hESC‐RPE‐based therapy for AMD.

## Graphical abstract

We generated immune‐tolerant mature hESC‐RPE cells by overexpressing PD‐L1. PD‐L1‐expressing hESC‐RPE grafts exhibited significantly higher survival, reduced apoptosis and enhanced visual protection in retinal degeneration models, which presents an alternative target to improve the efficiency of hESC‐RPE‐based therapy for AMD.

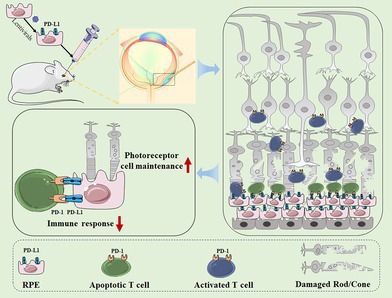

## 1 Introduction

Age‐related macular degeneration (AMD), prevalent among individuals over 55, is a progressive retinal disorder that primarily impacts the macula and can result in severe central vision loss and diminished quality of life [1]. The global prevalence of AMD is projected to reach 288 million by 2040 [2]. Although there is no cure, early treatment can slow AMD progression and alleviate symptoms. Current treatments include nutritional supplements such as antioxidant vitamins and minerals, anti‐VEGF therapies, photodynamic therapy, laser photocoagulation and intravitreal injections of complement pathway inhibitors [3, 4, 5, 6]. However, due to the loss of retinal pigment epithelium (RPE) and photoreceptors, cell replacement strategies are essential to prevent central vision loss. Numerous clinical studies have demonstrated that RPE suspension or sheet transplantation is safe and feasible for AMD patients [7, 8, 9, 10, 11]. Allogeneic human pluripotent stem cell (hPSC) derived RPE has become the primary cell source for transplantation [12]. However, a mismatch of major histocompatibility complex (MHC) between donor and recipient poses a risk of transplant rejection. For example, McGill et al. reported that allogeneic RPE grafts failed in the subretinal space of nonhuman primates, likely due to immune rejection [13]. Moreover, severe intraocular inflammation often associated with AMD, particularly wet AMD, of which the blood‐retinal barrier is impaired, may further increase the risk of graft rejection [14, 15, 16, 17]. Therefore, it is more urgent to improve the survival of RPE grafts in wet AMD.

Immune‐engineered hPSC has been studied to achieve immune evasion [18]. Many of these strategies include ablating MHC‐I and ‐II genes and overexpressing immune checkpoints [19, 20, 21, 22]. These approaches involved editing multiple genes to interfere with different immune cells across the innate and adaptive aspects of the immune system regardless of the type of donor cells. The application in specially differentiated derivatives has also shown promises, such as co‐knockout of MHC‐I, CD155 and B7‐H3 in induced pluripotent stem cell (iPSC) derived insulin‐secreting β cells and expression of PD‐L1 and CTLA4Ig in human embryonic stem cell (hESC) derived hepatocyte‐like cells, promoting immune tolerance [23, 24]. Extensive immune genetic modifications are undesirable in RPE because RPE cells endogenously express some immunomodulatory factors playing a role in immunosuppression [25]. No exploration of inducing immune tolerance of RPE grafts to the inflammatory milieu in the degenerative retina was observed.

We sought to transplant hESC‐RPE cells in retinal degenerative rodent models and screened immune inhibitory targets. We found that PD‐1 accumulated in the rejected retina. The PD‐1/PD‐L1 axis is a key pathway to induce T cell apoptosis [26]. Moreover, PD‐L1 also plays an important role in maintaining ocular immune privilege via suppressing inflammatory cytokines produced by activated T cells [27, 28, 29]. In our results, hESC‐RPE grafts overexpressing PD‐L1 showed increased survival and improved visual protection in rodent models. Therefore, PD‐L1 overexpression is a promising strategy for promoting RPE graft tolerance in inflamed retinas of wet AMD.

## 2 Materials and Methods

### 2.1 Human Embryonic Stem Cell (hESC) Culture and Differentiation Into RPE

The Q‐CTS‐hESC‐2 cell line was provided by the State Key Laboratory of Stem Cells and the Reproductive Biology, Institute of Zoology, Chinese Academy of Sciences and was tested and authenticated by the Chinese National Institutes for Food and Drug Control (Report number SH201402035) [30]. hESC line Q‐CTS‐hESC‐2 was cultured in xeno‐free Essential 8 Medium (Gibco, A1517001) as previous described [31]. The differentiation of hESC into RPE cells was performed according to the reported protocol in our laboratory [32]. Briefly, hESCs were induced with differentiation medium until the diameter of the pigment foci reached at least 1 mm and then picked out and placed in a 6‐well plate pre‐coated with recombinant human vitronectin‐N/rhVTN‐N (Gibco, A14700), described as passage 0 of hESC‐RPE cells. The hESC‐RPE cells were digested with TrypLE Express (Gibco, 12605028) and passaged every 3 weeks at a 1:3 split ratio. We froze hESC‐RPE cells at passage 1 with 90% CTS KnockOut SR XenoFree +10% DMSO. The hESC‐RPE cells at passages 3–5 were cultured with the differentiation medium and used for experiments. The differentiation medium consisted of CTS KnockOut DMEM (Gibco, A1286101), 20% CTS KnockOut SR XenoFree (Gibco, 12618012), 1% MEM NEAA Non‐Essential Amino Acids (Gibco, 11140‐050), 1% CTS GlutaMAX‐I (Gibco, A12860‐01), 1‰ β‐mercaptoethanol (Gibco, 21985023), 100 U/mL penicillin, and 100 mg/mL streptomycin (Sigma Aldrich, V900929).

### 2.2 PBMC Culture

Human peripheral blood mononuclear cells (hPBMCs) (Novobiotec, 932057) were cultured in PBMC medium containing RPMI 1640 (Sigma Aldrich, R8758) supplemented with 10% Fetal Bovine Serum (Clark Bioscience, FB25015), 1% penicillin/streptomycin.

### 2.3 Animals

RCS, RCS‐rdy^+^ (control) rats, and RD10 mice were provided by the Experimental Animal Center of Third Military Medical University (Army Medical University). Animals were housed in specific pathogen‐free conditions under 12‐h light–dark cycles in the Animal Care Center of Southwest Hospital. All animal experiments were approved by the Committee of Animal Care of the Third Military Medical University (AMUWEC20197039).

### 2.4 Transduction With PD‐L1 Overexpression Lentiviruses in hESC‐RPE

The hESC‐RPE cells were seeded into 6/24‐well plates at 1 × 10^5^ cells/cm^2^ density. Then, hESC‐RPE cells were incubated with lentiviral particles encoding PD‐L1 (OBiO, custom product) tagged with EGFP at a multiplicity of infection of 1 in differentiation medium with 5 μg/mL polybrene for 16 h. The hESC‐RPE cells infected with the EGFP lentiviral particles were used as a negative control. Subsequently, the medium was replaced with fresh medium, and cells were cultured for 48–72 h. Transduction efficiency was analysed through EGFP expression by fluorescence microscopy and flow cytometry.

### 2.5 Immunofluorescence Staining

For cell immunofluorescence staining, hESC‐RPE cells grown on coverslips were washed twice with PBS and fixed in 4% paraformaldehyde (PFA) at room temperature for 15 min. Then, cells were blocked with 10% goat serum, 1% bovine serum albumin (BSA) and 0.5% Triton X‐100 at 37°C for 30 min. The primary antibodies were diluted to work concentrations in antibody dilution buffer (1 × PBS, 3% goat serum, 1% BSA and 0.5% Triton X‐100) and incubated at 4°C overnight. The following primary antibodies were used: rabbit anti‐human PD‐L1 (1:500, Abcam, ab205921), mouse anti‐RPE65 (1:50, Santa Cruz, sc‐390787) and rabbit anti‐ZO‐1 (1:100, Proteintech, 21773‐1‐ap). After washing with PBS three times, cells were incubated with secondary antibodies of goat anti‐rabbit IgG (H + L) conjugated with Alexa Fluor 568 (1:500, Invitrogen, A‐11011) or goat anti‐mouse IgG (H + L) conjugated with Alexa Fluor 568 (1:500, Invitrogen, A‐11004) at 37°C for 1 h. And cell nuclei were stained with 4′, 6‐diamidino‐2‐phenylindole (DAPI) for 30 s. Coverslips dripping with anti‐fluorescent quencher were covered on glass slides. Images were visualised using Zeiss LSM880 laser confocal microscopy.

For tissue immunofluorescence staining, eyes were enucleated immediately following euthanasia by carbon dioxide inhalation at 1, 2 and 4 weeks post‐transplantation. Eyes were fixed in 4% PFA for 2 h at 4°C, dehydrated in 30% glucose solution overnight, and embedded in the optimum cutting temperature (OCT) compound for freeze‐sectioning. The frozen sections were mounted onto adhesive‐coated glass slides and underwent the same staining procedure as cell immunofluorescence. Primary and secondary antibodies used in this study were: rabbit anti‐human PD‐L1 (1:500, Abcam, ab205921), rabbit anti‐human MTCO2 (1:200, Abcam, ab79393), rabbit anti‐human BEST1 (1:200, Abcam, ab14929), mouse anti‐Rhodopsin (1:200, Abcam, ab5417), mouse anti‐rat CD3 (1:100 BD Biosciences, 550295), mouse anti‐rat CD161a (1:100 BD Biosciences, 555006), rabbit anti‐CD79a (1:200, Abcam, ab300150), rabbit anti‐Iba1 (1:500, Wako, 019–19741), goat anti‐rabbit IgG (H + L) conjugated with Alexa Fluor 568 (1:500, Invitrogen, A‐11011), goat anti‐mouse IgG (H + L) conjugated with Alexa Fluor 568 (1:500, Invitrogen, A‐11004), goat anti‐mouse IgG (H + L) conjugated with Alexa Fluor 647 (1:500, Beyotime, A0473).

### 2.6 Flow Cytometry Analysis

Retinas from RCS and RCS‐rdy^+^ rats were harvested and dissociated into single cells using sCelLiveTM tissue dissociation solution (Singleron Biotechnologies, 1020066). Single retinal cells were stained in staining buffer (PBS + 2% FBS) with cell surface markers CD45 (Pacific Blue anti‐rat CD45, Biolegend, 202225), CD3 (APC anti‐rat CD3, Biolegend, 201413), CD4 (APC/Cyanine7 anti‐rat CD4, Biolegend, 201518), CD8 (PerCP anti‐rat CD8a, Biolegend, 201712) and/or CD161 (PE/Cyanine7 anti‐rat CD161, Biolegend, 205610) for 30 min at 4°C. Cells were then washed twice and resuspended in 300 μL staining buffer. Samples were captured on the BD LSRFortessa, and results were analysed using FlowJo 10.8.1 (BD Biosciences). Staining for hESC‐RPE cells or hPBMCs was the same as for retinal cells; the corresponding antibody details are listed in the cell experiment.

### 2.7 In Vitro Cytotoxicity Assay

In vitro cytotoxicity assay was performed according to the previous study [20]. Twenty thousand hESC‐RPE cells were plated in a well of a 96‐well plate and transduced with PD‐L1 overexpressing or control lentivirus. After 72 h, infected hESC‐RPE cells were cocultured with hPBMCs (1:10) in the PBMC medium containing 100 U/mL rhIL‐2 (Peprotech, AF‐200‐02). The hPBMCs were activated by anti‐human CD3/OKT3 (Biolegend, 317325) and anti‐human CD28 (Biolegend, 302933) antibodies before the coculture. After a 3‐day‐coculture, supernatants were collected and analysed via CyQUANT LDH cytotoxicity assay (Invitrogen, C20300) according to the manufacturer's instructions. PBMC medium was used as a background control. hPBMCs or RPE cells cultured alone were negative controls for LDH release. Lysis buffer‐treated RPE cells were used as a positive control for LDH release.

### 2.8 T‐Cell Proliferation Assay

hESC‐RPE cells were seeded in 24‐well plates and transduced with PD‐L1 overexpressing or control lentivirus. After 72 h, infected hESC‐RPE cells were cocultured with hPBMCs at the same ratio as the cytotoxicity assay. Before coculture, hPBMCs were activated with anti‐human CD3/CD28 antibodies and labelled with CellTrace Violet (Invitrogen, C34571) following the manufacturer's instructions. After a 5‐day‐coculture, hPBMCs were collected and stained with anti‐CD4 (Biolegend, 317415) and CD8 (Biolegend, 344707) antibodies. Then, they were analysed on a BD LSRFortessa for Violet intensity. The hPBMCs cultured alone were used as a negative control.

### 2.9 T‐Cell Activation Assay

hESC‐RPE cells were cocultured with non‐activated hPBMCs for 3 days at the same ratio as the T‐cell proliferation assay. The hPBMCs were stained with anti‐CD4, anti‐CD8 and anti‐CD69 (Biolegend, 310913) (T‐cell activation marker) antibodies and analysed on a BD LSRFortessa. Cultured alone, hPBMCs served as a negative control, whereas those activated with anti‐CD3/CD28 antibodies were a positive control.

### 2.10 T‐Cell Apoptosis Assay

T cells were isolated from hPBMCs using MojoSort Human CD3 T Cell Isolation Kit (Biolegend, 480131) according to the manufacturer's instructions. hESC‐RPE cells were cocultured with activated T cells (activated by CD3/CD28) for 3 days at the ratio of 1:5 (hESC‐RPE: T cell). T cells were stained with PI and Hoechst (C1056, Beyotime) for 20 min at 4°C. Then, they were analysed on a BD LSRFortessa.

### 2.11 Subretinal Transplantation

CTL hESC‐RPE (control) or PD‐L1^+^ hESC‐RPE cells were dissociated into single cells using TrypLE Express. Cells were centrifuged at 1000 rpm for 5 min and resuspended in sterile PBS (on ice) to a final concentration of 100,000 cells/μL before transplantation.

At postnatal day 35 (RCS rats) or day 25 (RD10 mice), animals were anaesthetised with intraperitoneal pentobarbital sodium (1.5% [wt/vol], 0.2 mL/100 g), and pupils were dilated with tropicamide phenylephrine eye drops (Santen Pharmaceutical Co. Ltd. Japan). Cells were transplanted into both eyes using a Hamilton syringe (33‐gauge needle) through sclera puncture. Rats received 200,000 cells/eye (2 μL), and mice 100,000 cells/eye (1 μL). PBS‐injected animals served as sham controls. No immunosuppression was given. A total of 105 rats (31 rats in the PBS group, 42 rats in the CTL hESC‐RPE group, 32 rats in the PD‐L1^+^ hESC‐RPE group) and 30 mice (10 mice in each group above) underwent transplantation. We performed visual function, morphological and immunological analysis on rats at 2 days, 1, 2, 4 and 8 weeks post‐transplantation. Visual function and morphology were also assessed in mice 1 week post‐transplantation. We sequenced the retinal single‐cell transcriptomes of rats transplanted with CTL and PD‐L1^+^ hESC‐RPE at 1 week post‐transplantation.

### 2.12 Scotopic Electroretinogram Recording

As previously described, the scotopic electroretinogram (ERG) recording was performed at 1, 2, 4 and 8 weeks post‐transplantation [33]. Briefly, rodents underwent 16 h of dark adaption before ERG testing. They were then anaesthetised with 1.5% pentobarbital sodium and dilated their pupils with tropicamide phenylephrine eye drops. Contact lens electrodes were placed on both corneas as recording electrodes, with reference and ground electrodes placed in the mouth and tail, respectively. Light stimulation densities were applied via a Stimulator (Mayo Corporation, Aichi, Japan). The amplitudes of a/b waves were recorded by a RETI‐Port device (Roland Consult) from 0.0001 to 10.0 cd*m/s^2^. Experiments were conducted in a dark room under dim red light.

### 2.13 Quantitative Polymerase Chain Reaction (qPCR) Analysis

Total RNA was extracted from hESC‐RPE/hPBMCs or rat retinal tissues 1 week post‐transplantation using the TRIZOL reagent (Takara, 9108) according to the manufacturer's instructions. The cDNA was synthesised with PrimeScriptTM RT reagent Kit with gDNA Eraser (Takara, RR047) following the manufacturer's protocol. The qPCR experiment was performed using the SYBRPrime qPCR Set (Bioground Biotech, BG0014) on the CFX96 Real‐time PCR Thermocycler (Bio‐Rad). Relative gene expression levels were normalised with the 2^−ΔΔCT^ method, and *Gapdh* or *β‐ACTIN* was used as the housekeeping gene. The primer sequences for qPCR are listed in Table S1.

### 2.14 Sample Dissociation and single‐cell RNA Sequencing (scRNA‐Seq)

Six RCS rats transplanted with CTL hESC‐RPE and six with PD‐L1^+^ hESC‐RPE were euthanized at 1 week post‐transplantation. All the eyeballs were collected and thoroughly washed with PBS. The anterior segments were removed, and the neuroretina was carefully extracted and placed in 500 μL PBS. The retinas were dissociated with sCelLiveTM tissue dissociation solution at 37°C for 5–10 min, and the dissociated cells were filtered with a 40 μm cell strainer. Then, the single cells were centrifuged at 300× g for 5 min, and the pellet was resuspended in 1 mL staining buffer (PBS + 2% FBS). Since it is challenging to capture transplanted RPE cells in the retinal single‐cell suspension, to ensure that the sample contains both transplanted cells and host retinal cells, a portion of the retinal single‐cell suspension was drawn for transplanted RPE sorting. Then, we mixed it with the remaining retinal single‐cell suspension. Single‐cell suspensions (2 × 10^5^ cells/mL) in PBS were loaded onto a microwell chip using the Singleron Matrix Single Cell Processing System. Barcoding Beads were collected from the microwell chip, followed by reverse transcription of the mRNA captured by the Barcoding Beads to obtain cDNA. PCR amplification was performed afterward. The amplified cDNA was then fragmented and ligated with sequencing adapters. The scRNA‐seq libraries were constructed according to the protocol of the GEXSCOPE Single Cell RNA Library Kits (Singleron) [34]. Individual libraries were diluted to 4 nM, pooled, and sequenced on Illumina novaseq 6000 with 150 bp paired‐end reads.

### 2.15 The Primary Analysis of Raw Read Data

Raw reads from scRNA‐seq were processed to generate gene expression profiles using CeleScope v1.15.0 (Singleron Biotechnologies) with default parameters. Briefly, Barcodes and UMIs were extracted from R1 reads and corrected. Adapter sequences and poly‐A tails were trimmed from R2 reads, and the trimmed R2 reads were then aligned to the custom reference genome containing the concatenated genomes of humans and Rattus using STAR (v2.6.1b) as described [35]. Only the percentage of cells with human transcripts was more than 80%, the cell was classified as human cells; If the percentage of human transcripts was less than 20%, the cell was classified as rattus cells. Uniquely mapped reads were assigned to exons with FeatureCounts (v2.0.1). Successfully assigned reads with the same cell barcode, UMI and gene were grouped to generate the gene expression matrix for further analysis.

### 2.16 scRNA‐Seq Data Analysis

The data were processed using the R package Seurat (v4.2.1) under R4.3.3. And low‐quality cells with < 200 measured genes and a high percentage of mitochondrial DNA contamination (> 15%) and haemoglobin gene (> 1%) were filtered out. The data were normalised, and then we integrated the datasets and applied batch correction using the MNN method. Each dataset's 4000 most variable genes were identified. Next, we selected the top 15 principal components to perform Uniform Manifold Approximation and Projection (UMAP) to embed the dataset into two dimensions. FindAllMarkers was used to find the marker genes of each cluster. We filtered significant positive genes expressed in > 25% of cells and logFC threshold > 0.25 using the Wilcoxon rank‐sum test. Then, we picked cluster 12, which represents human genes, as the transplanted hESC‐RPE cells. And other cells were picked out as the cells of RCS retinas. Then, UMAP clustering analysis is performed in the above way. Cluster information of the UMAP clustering group was manually annotated using the known markers. The differential expressed genes (DEGs) analysis was performed using FindMarkers in the Seurat package. DEGs were recognised by the adjusted *p* value < 0.05 and avg_log2FC > 0.1 or < −0.1. GO and KEGG enrichment of DEGs were performed by clusterProfiler (v4.6.2) R package.

For the scRNA‐seq data published in the GEO database, single‐cell transcriptomic data with accession number GSE212896 was used to analyse the alteration of the pathway for hESC‐RPE or hiPSC‐RPE after transplantation. The quality control was performed as previously described [36]. Then the data were normalised and applied batch correction. The unsupervised clustering analysis, and cell clustering definition were also conducted as previously described [36]. According to the literature [36], RPE cells tended to mature more after transplantation. To accurately analyse the changes in the gene expression and most critical pathways after RPE transplantation, we selected mature RPE cells for subsequent analysis. DEG analysis was done as above, and the GSEA analysis was performed using the clusterProfiler (v4.6.2) R package.

### 2.17 Statistical Analysis

All results were presented as mean ± SEM and analysed by GraphPad Prism. Dataset normality was assessed using the Shapiro–Wilk test. The statistical significance between the two groups was determined using an unpaired Student's *t*‐test and Welch's *t*‐test, as appropriate. The Mann–Whitney test was used for non‐normally distributed datasets. One‐way ANOVA followed by Tukey's post hoc multiple comparisons test was performed for multiple groups. Two‐way ANOVA followed by Sidak's post hoc multiple comparisons test was used for groups with two variables. *p* < 0.05 was considered significant.

## 3 Results

### 3.1 The Expression of PD‐1 in the Rat Retina is Elevated Following hESC‐RPE Transplantation

**FIGURE 1:**
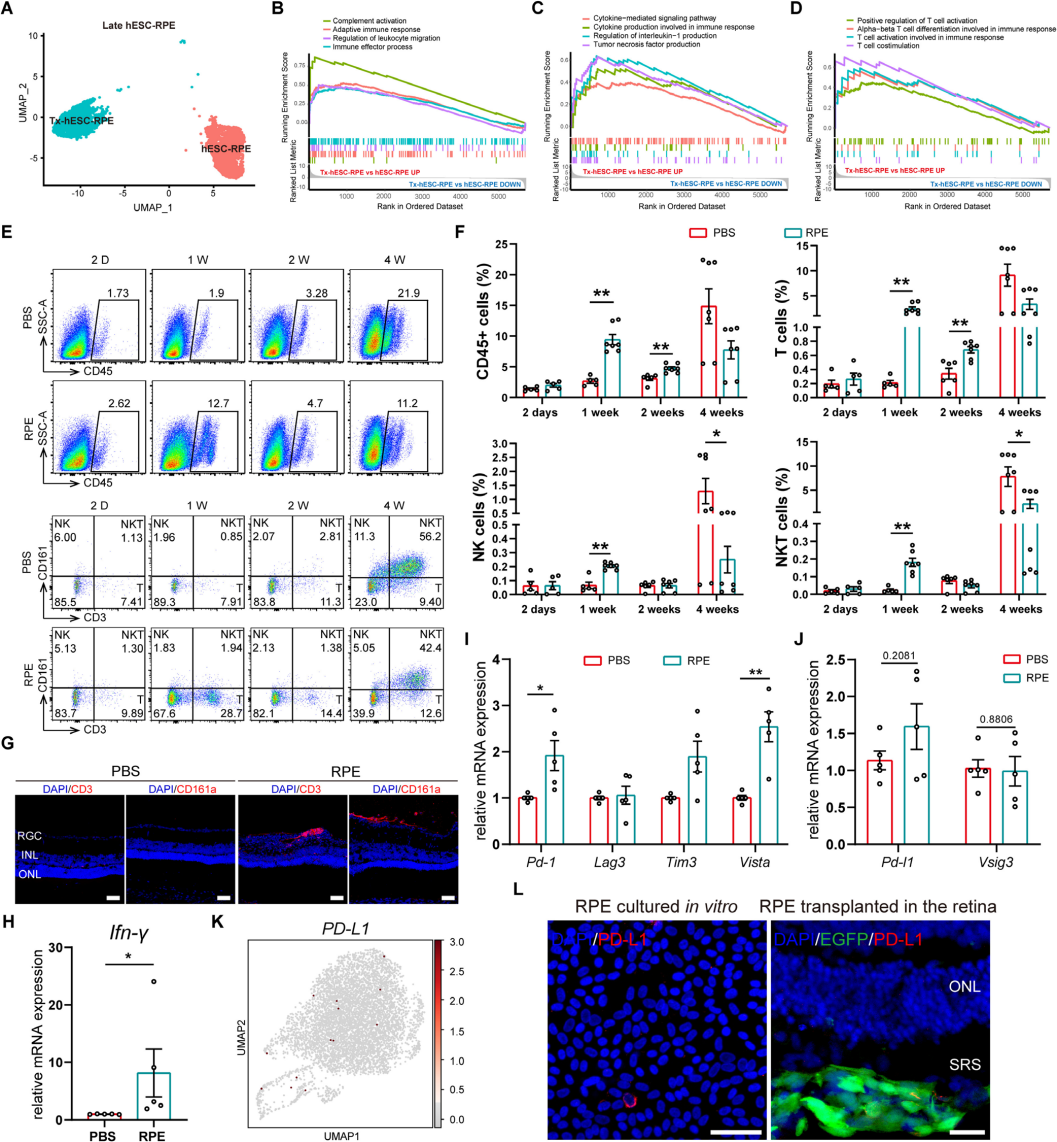
Immune response characteristics and immune checkpoints changes in RCS rat retina after hESC‐RPE transplantation. (A) UMAP plot of late (in red) versus transplanted (in green) hESC‐RPE cells. Tx‐hESC‐RPE, the transplanted hESC‐RPE. (B–D) The GSEA analysis of the immune‐related (B), cytokine‐related (C) and T cell‐related pathways (D) for Tx‐hESC‐RPE versus late hESC‐RPE. The single‐cell transcriptomic data were downloaded from the public database (GEO database: GSE212896). (E) The representative images of flow cytometry analysis of immune cells (CD45^+^), T cells (CD3^+^), NK cells (CD3^−^CD161^+^) and NKT cells (CD3^+^CD161^+^) in hESC‐RPE or PBS (control) engrafted‐retinas at 2 days, 1, 2, 4 weeks after transplantation. (F) The quantification of CD45^+^ immune cells, T cells, NK cells and NKT cells in hESC‐RPE or PBS (control) engrafted‐retinas at 2 days, 1, 2, 4 weeks after transplantation, *n* = 5–7 retinas. Mann‐Whitney test. Data presented as mean ± SEM; *p < 0.05, **p < 0.01. (G) Immunofluorescence images of T cells (CD3) and NK cells (CD161a) in the vitreous and retinas of hESC‐RPE‐ or PBS‐engrafted eyes at 1 week post‐transplantation. Scale bar, 50 μm. (H) The expression of *Ifn‐γ* in PBS‐ and hESC‐RPE‐engrafted retinas at 1 week post‐transplantation, as measured by qPCR, *n* = 5 retinas. Mann‐Whitney test. Data presented as mean ± SEM; *p < 0.05. (I) The expression of immune checkpoint receptors *Pd‐1*, *Lag3*, *Tim3*, *Vista* in PBS‐ and hESC‐RPE‐engrafted retinas at 1 week post‐transplantation by qPCR, *n* = 5 retinas. Welch's *t*‐test. Data presented as mean ± SEM; *p < 0.05, **p < 0.01. (J) The expressions of *Pd‐l1* and *Vsig3* in PBS‐ and hESC‐RPE‐engrafted retinas at 1 week post‐transplantation by qPCR, *n* = 5 retinas. Unpaired Student'*t*‐test. Data presented as mean ± SEM. (K) The endogenous expression of *PD‐L1* in hESC‐RPE cells at the single‐cell transcriptomic level. (L) Immunofluorescence showing PD‐L1 expression in hESC‐RPE cells cultured in vitro (left, scale bar 50 μm) and transplanted in the retina (right, scale bar 20 μm). EGFP: Transplanted hESC‐RPE. ONL: outer nuclear layer; SRS: subretinal spaces.

To understand the changes in immune‐related pathways in RPE cells after transplantation, we analysed single‐cell transcriptomic data from the public GEO database (GSE212896 [36]), focusing on human iPSC and ESC derived RPE cells transplanted into rabbits' subretinal spaces (SRS) as sheets for 1 month. The quality control, unsupervised clustering analysis, and cell clustering definition were performed as previously described [36]. Parikh BH et.al's research showed that the characteristics of transplanted RPE cells aligned more closely with late‐stage RPE cultured in vitro [36], so we just performed clustering and comparative analyses on transplanted (Tx‐hESC‐RPE or Tx‐hiPSC‐RPE) and age‐matched late RPE cells (hESC‐RPE or hiPSC‐RPE) to minimise the influence of early‐stage RPE. GSEA analysis showed activation of immune‐related pathways post‐transplantation for both hiPSC‐RPE and hESC‐RPE, with significant enrichment in pathways involved in T cell differentiation, activation and costimulation (Figures 1A–D and S1).

Next, we examined the T cell response process following hESC‐RPE transplantation into RCS rats, a model of retinal degeneration. We assessed T cell changes in the retina at the early (postnatal day 20), middle (postnatal day 40) and late (postnatal day 60) stage of degeneration. The result indicated a progressive increase in T cells in the retina as degeneration advanced (Figure S2), with higher risks of immune rejection when the later hESC‐RPE was transplanted. We injected hESC‐RPE into RCS rats at the middle stage of degeneration, using PBS as a control. Flow cytometry analysis revealed no increase in immune cells 2 days post‐transplantation compared with PBS control. However, CD45^+^ cells and CD3^+^ T cells rose markedly at 1 week and remained elevated through 2 weeks post‐transplantation. NK cells (CD3^−^CD161^+^) and NKT cells (CD3^+^CD161^+^) increased at 1 week but decreased at 2 weeks, showing no significant difference from the PBS control (Figure 1E,F). Notably, at 4 weeks post‐transplantation, the proportion of immune cells in PBS control exceeded that in the RPE group with a significant difference in NK and NKT cells. It was probably due to aggravated retinal inflammation in RCS rats at the advanced degeneration stage, as shown before (Figure S2), and RPE grafts part of relieved this inflammatory progression. Increased T cells and NK cells infiltrated the vitreous and inner retina (Figure 1G), and the inflammatory cytokine *Ifn‐γ* significantly upregulated at the mRNA level after hESC‐RPE transplantation (Figure 1H). These findings indicated rapid and intense T cell responses associated with hESC‐RPE rejection.

To identify immune checkpoints that could enhance immune tolerance of hESC‐RPE grafts, we examined the expression of T cell‐associated immune checkpoint genes in rejected retinas after hESC‐RPE transplantation [26, 37, 38, 39]. The immune checkpoint receptor molecules *Pd‐1* and *Vista* were significantly upregulated (Figure 1I), while the expression of *Pd‐l1* (*Pd‐1* ligand) and *Vsig3* (*Vista* ligand) did not increase reactively (Figure 1J). Given the high expression of Vista in naïve T cells and regulatory T cells (T reg) [39], the upregulation of Vista may reflect the natural immunosuppression properties of hESC‐RPE cells rather than immune activation. In addition, we also observed upregulated *PD‐1* in activated hPBMCs (Figure S3). Therefore, we chose the PD‐1/PD‐L1 axis as our immune‐tolerant strategy for RPE in this study. We performed a single‐cell transcriptome analysis of mature hESC‐RPE cells cultured in vitro and found that our hESC‐RPE cells expressed negligible *PD‐L1* at the mRNA level (Figure 1K). Meanwhile, hESC‐RPE hardly expressed PD‐L1 at the protein level, even after transplantation into SRS (traced with EGFP) (Figure 1L). Furthermore, we also observed that the expression of *PD‐L1* in Tx‐hESC‐RPE or Tx‐hiPSC‐RPE was not significantly higher than that before transplantation into rabbits (Figure S4).

### 3.2 PD‐L1 Overexpression Promotes hESC‐RPE Survival Under Inflammatory Conditions In Vitro

**FIGURE 2:**
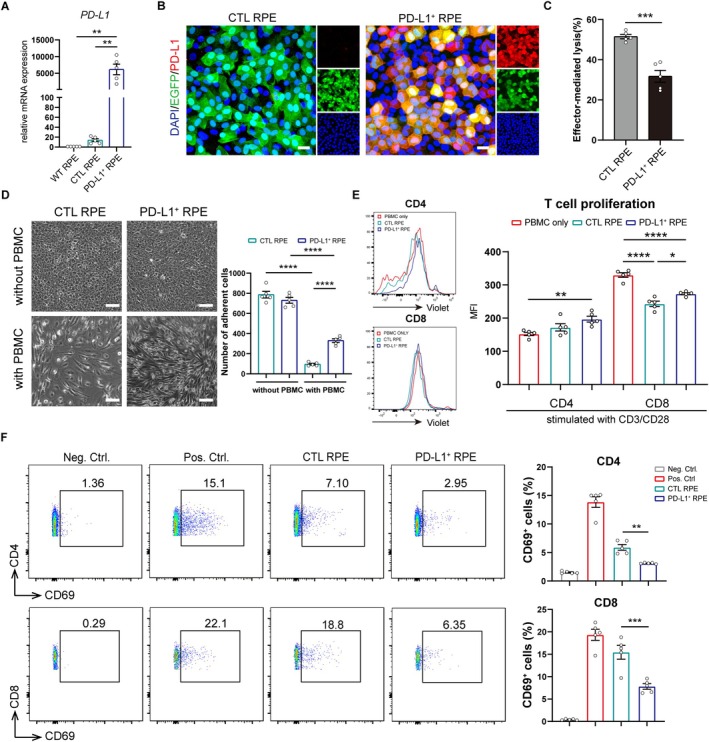
PD‐L1^+^ hESC‐RPE evades killing when cocultured with allogeneic hPBMCs in vitro. (A) The expression of *PD‐L1* in hESC‐RPE transduced with PD‐L1 overexpression or empty vector lentiviruses, as measured by qPCR, *n* = 5 independent experiments. One‐way ANOVA followed by Tukey's post hoc test. Data presented as mean ± SEM; **p < 0.01. (B) Immunofluorescence showing PD‐L1 expression in control (CTL) or PD‐L1^+^ RPE. Scale bar, 20 μm. (C) The bar graph showing the percentage of hPBMCs cytotoxicity against CTL and PD‐L1^+^ RPE cells at the cocultured ratio of 10:1 (PBMC: RPE), *n* = 5 donors. Unpaired Student's *t*‐test. Data presented as mean ± SEM; ***p < 0.001. (D) The morphology and survival of CTL and PD‐L1^+^ RPE after coculturing with hPBMCs at the ratio of 10:1 (PBMC: RPE). Scale bar, 100 μm. The bar graph represents the number of adherent hESC‐RPE cells. *n* = 5 donors. Two‐way ANOVA followed by Sidak's post hoc test. Data presented as mean ± SEM; ****p < 0.0001. (E) Flow cytometry histogram of the proliferation in CD4 and CD8 T cell populations after cocultured with CTL or PD‐L1^+^ RPE (left); the statistical graph of the mean fluorescence intensity (MFI) of Violet (right). hPBMCs cultured alone were used as the negative control. hPBMCs were pre‐stimulated with anti‐CD3/CD28 antibodies. *n* = 5 donors. One‐way ANOVA followed by Tukey's post hoc test. Data presented as mean ± SEM; *p < 0.05, **p < 0.01, ****p < 0.0001. (F) The percentage of CD69^+^ cells in CD4 (up) and CD8 (down) T cell populations when cocultured with CTL or PD‐L1^+^ RPE. hPBMCs cultured alone were used as the negative control. hPBMCs stimulated with CD3/CD28 were used as the positive control. *n* = 5 donors. One‐way ANOVA followed by Tukey's post hoc test. Data presented as mean ± SEM; **p < 0.01, ***p < 0.001.

To examine whether PD‐L1 overexpression could protect hESC‐RPE from immune cell attack, we constructed a lentiviral vector to overexpress PD‐L1 tagged with EGFP under the CMV promotor. The experimental group (PD‐L1^+^ hESC‐RPE) consisted of hESC‐RPE infected with PD‐L1‐EGFP lentiviruses, while the control group (CTL hESC‐RPE) included cells infected with the EGFP. Both transfection groups displayed prominent green fluorescence (Figure S5A), with over 90% transfection efficiency compared to wild‐type non‐infected hESC‐RPE by flow cytometry (Figure S5B). *PD‐L1* expression was significantly upregulated in PD‐L1^+^ hESC‐RPE compared to CTL or WT cells (Figure 2A), and localised mainly to the cell membranes (Figure 2B). Additionally, PD‐L1 overexpression did not alter the expression of mature hESC‐RPE markers, *RPE65*, *BEST1* and *CRALBP*, or tight junction formation (Figure S5C,D).

To investigate if PD‐L1 overexpression is sufficient to protect hESC‐RPE from T cell cytotoxicity, we cocultured CTL or PD‐L1^+^ hESC‐RPE with hPBMCs for 3 days. Lactate dehydrogenase (LDH) release, an indicator of cell death, was significantly lower in PD‐L1^+^ hESC‐RPE than in CTL hESC‐RPE (Figure 2C), indicating enhanced survival of PD‐L1^+^ hESC‐RPE in inflammatory conditions. The cytotoxicity of hPBMCs led to the shedding and apoptosis of hESC‐RPE cells, and bright‐field observation also showed that there was more adherent PD‐L1^+^ hESC‐RPE cells than adherent CTL hESC‐RPE cells after coculture with hPBMCs (Figure 2D). The protective effect of PD‐L1 may involve T‐cell inhibition. We cocultured CellTrace Violet‐prelabeled hPBMCs with PD‐L1^+^ or CTL hESC‐RPE for 5 days and measured Violet mean fluorescence intensity (MFI) to test T cell proliferation. Lower Violet MFI represented more T cell proliferation. PD‐L1 significantly inhibited CD8^+^ T cell proliferation but not CD4^+^ T cells compared with CTL RPE group (Figure 2E). Additionally, PD‐L1^+^ hESC‐RPE reduced the T cell activation marker (CD69) in both CD4^+^ and CD8^+^ T cells after a 3‐day coculture with hPBMCs. (Figure 2F). These findings suggest that PD‐L1 primarily inhibits CD8^+^ T cells in vitro, aligning with prior reports [40]. Interestingly, human leukocyte antigen (HLA) gene *HLA‐A* (HLA I molecule) and *HLA‐DR* (HLA II molecule) were downregulated in PD‐L1^+^ hESC‐RPE after coculture with hPBMCs compared with CTL RPE (Figure S6). It is known that the expression of HLA I/II in RPE could be upregulated by some inflammatory cytokines, especially IFN‐γ [41]. We speculated that PD‐L1 inhibited T cell‐mediated immune responses and reduced the accumulation of inflammatory cytokines. Correspondingly, the stimulating upregulation of HLA‐A/DR expression in PD‐L1^+^ hESC‐RPE was reduced. To further verify the effect of PD‐L1 on T cell apoptosis, we cocultured activated T cells with CTL/PD‐L1^+^ hESC‐RPE. PD‐L1^+^ hESC‐RPE significantly promoted the apoptosis of activated T cells compared with CTL RPE (Figure S7).

### 3.3 PD‐L1 Overexpression Enhances hESC‐RPE Graft Survival and Visual Preservation in Retinal Degeneration Models

**FIGURE 3:**
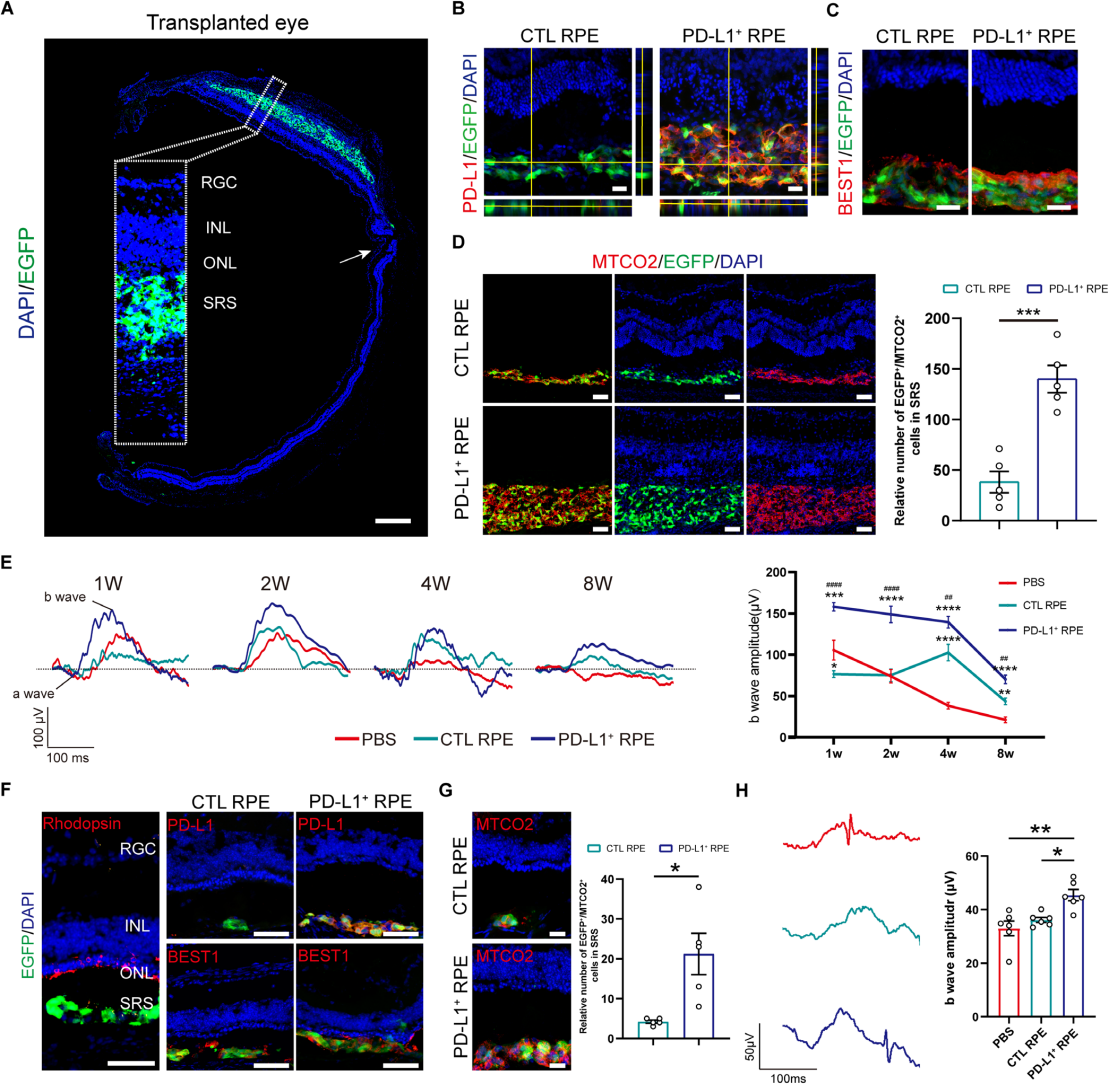
PD‐L1^+^ hESC‐RPE exhibited better survival and visual protection in two retinal degenerative rodents. (A) The EGFP^+^ grafts in the retina of RCS rats at 1 week post‐operation by confocal mosaic image analysis. The arrow showed the injection site. Scale bar, 500 μm. (B) Orthogonal projection of PD‐L1 staining in CTL RPE‐ and PD‐L1^+^ RPE‐engrafted retina of rats at 1 week post‐transplantation. Scale bar, 20 μm. (C) BEST1 immunofluorescence images of CTL RPE grafts and PD‐L1^+^ RPE grafts. Scale bar, 20 μm. (D) The immunofluorescence of human‐specific marker MTCO2 in hESC‐RPE grafts (left, scale bar 50 μm) and statistical analysis of the relative number of EGFP^+^/MTCO2^+^ cells in the CTL RPE and PD‐L1^+^ RPE‐engrafted rat retinas (right) at 1 week post‐transplantation, *n* = 5 eyes. Unpaired Student's *t*‐test. Data presented as mean ± SEM; ***p < 0.001. (E) Representative ERG images (left) and b wave amplitudes quantification (right) of RCS rats in the PD‐L1^+^ RPE, CTL RPE and PBS groups at 1, 2, 4 and 8 weeks post‐transplantation. Stimulus light intensity was 3.0 cd*m/s^2^. *n* = 6–12 eyes. One‐way ANOVA followed by Tukey's post hoc test. Data presented as mean ± SEM; *p < 0.05, **/^##^ *p* < 0.01, ***p < 0.001, ****/^####^ *p* < 0.0001. (F) The EGFP^+^ grafts in the retina of RD10 mice at 1 week post‐transplantation (left) and the immunofluorescence images of PD‐L1, BEST1 (middle, right) in CTL RPE grafts or PD‐L1^+^ RPE grafts. Scale bar 50 μm. (G) The immunofluorescence images of EGFP^+^/MTCO2^+^ RPE grafts in the CTL and PD‐L1^+^ RPE groups at 1 week post‐transplantation (left, scale bar 20 μm). Statistical analysis of the relative number of EGFP^+^/MTCO2^+^ cells (right), *n* = 5 eyes. Welch's *t*‐test. Data presented as mean ± SEM; *p < 0.05. (H) Representative ERG images and b wave amplitudes quantification of RD10 mice in the PD‐L1^+^ RPE, CTL RPE and PBS groups at 1 week post‐transplantation. *n* = 6 eyes. One‐way ANOVA followed by Tukey's post hoc test. Data presented as mean ± SEM; *p  < 0.05, **p  < 0.01.

We initially transplanted PD‐L1^+^ or CTL hESC‐RPE into RCS rats, where grafts exhibited dense packing (Figure 3A). PD‐L1^+^ RPE grafts stably expressed PD‐L1 on the cell membrane (Figure 3B), and both graft types expressed the mature RPE marker BEST1 (Figure 3C). At 1 week post‐transplantation, coinciding with the peak of immune rejection, the number of living transplanted cells (co‐expressing EGFP and human mitochondrial marker MTCO2) was significantly higher in the PD‐L1^+^ RPE compared to CTL RPE (Figure 3D). Scotopic electroretinography (ERG) measurements were conducted at 1‐, 2‐, 4‐, and 8‐weeks post‐transplantation. Increased a/b wave amplitudes represented improved visual function. Our results showed PD‐L1^+^ RPE grafts conferred sustained visual function improvement up to 8 weeks post‐transplantation accompanied by significantly increasing b wave at 3.0 cd*m/s^2^, the amplitudes of which intensity reflects the rod‐cone responses, outperforming CTL RPE grafts and PBS group. CTL RPE also exploited visual protection compared with the PBS group, however, this visual improvement was a delayed response, only showing up at 4‐ and 8‐weeks post‐transplantation. Moreover, the visual function decreased in the CTL RPE group with a notably lower b‐wave amplitude at 1‐week post‐transplantation compared to PBS controls. We speculated that it was related to rapid immune responses after CTL RPE transplantation (Figure 3E). However, we did not find the difference in a wave amplitudes among the three groups (data not shown).

Similarly, we transplanted PD‐L1^+^ and CTL hESC‐RPE cells into RD10 mice, another model of retinal degeneration. At 1 week post‐transplantation, PD‐L1 was expressed on the cell membrane in PD‐L1^+^ RPE, and both graft types expressed BEST1 (Figure 3F). The number of transplanted cells (co‐expressing EGFP and MTCO2) was significantly higher in the PD‐L1^+^ RPE group than in the CTL RPE group (Figure 3G), with a corresponding increase in b wave amplitude in the PD‐L1^+^ group over the CTL and PBS groups (Figure 3H). These findings support PD‐L1's protective effect on RPE grafts and its role in promoting visual preservation in retinal degeneration models.

### 3.4 scRNA‐Seq Reveals Lower Immunogenicity and Higher Survival Signal in PD‐L1 ^+^ hESC‐RPE Grafts

**FIGURE 4:**
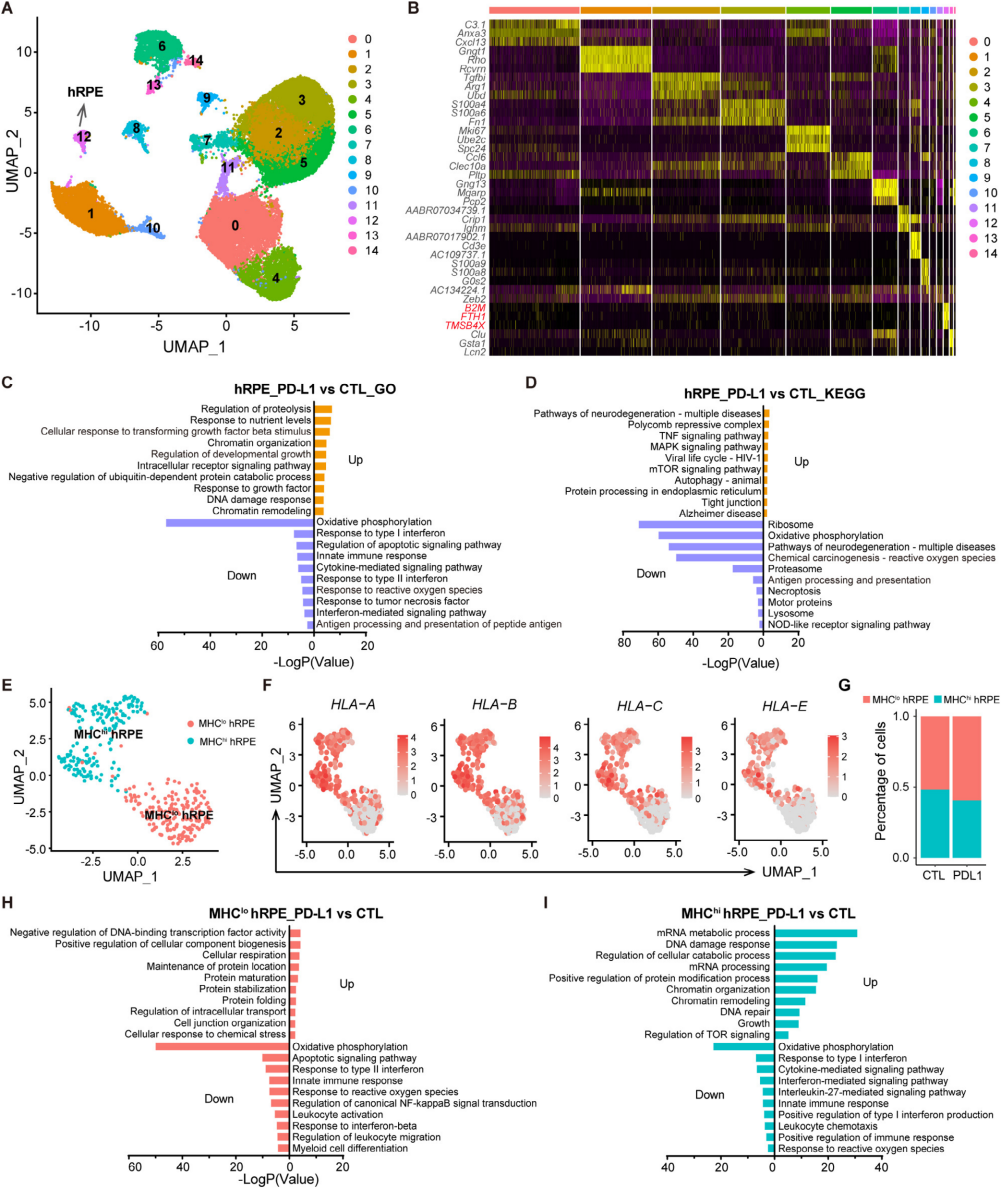
Single‐cell transcriptomic analysis of CTL and PD‐L1^+^ hESC‐RPE grafts 1 week post‐transplantation. (A) UMAP plot of single‐cell transcriptomics of CTL and PD‐L1^+^ RPE‐engrafted rat retinas. A total of 33,421 cells yielded 14 clusters with different colors. Each group contained 12 retinas from 6 rats. (B) Heatmap plot of the top three differentially expressed genes in each cell cluster. Human‐specific genes are highlighted in red. (C, D) GO (C) and KEGG (D) enrichment analysis of transplanted PD‐L1^+^ RPE versus CTL RPE. (E) UMAP plot of the hESC‐RPE grafts, categorised as MHC^hi^ (green) and MHC^lo^ (red). (F) The feather plot shows the gene expression of HLA I molecules (*HLA‐A, HLA‐B, HLA‐C, HLA‐E*) in the hESC‐RPE grafts. (G) The fraction of MHC^hi^ (green) and MHC^lo^ (red) cells in CTL RPE and PD‐L1^+^ RPE grafts. (H, I) GO enrichment analysis of PD‐L1^+^ RPE versus CTL RPE in MHC^hi^ and MHC^lo^ subpopulations.

To further assess PD‐L1's effect on hESC‐RPE grafts and host retinal cells, we performed scRNA‐seq on rat retinas engrafted with hESC‐RPE 1 week post‐transplantation. After quality control, we analysed 33,421 cells, comprising 10,610 cells from PD‐L1^+^ and 22,811 cells from CTL hESC‐RPE‐engrafted retinas. UMAP clustering identified 14 distinct cell subsets based on their expression profiles (Figure 4A). Each subset's top three differentially expressed genes (DEGs) were plotted in a heatmap (Figure 4B). Cluster 12 showed high expression of human‐specific genes such as *B2M*, *FTH1* and *TMSB4X* (Figure 4B). In addition, we found that these cells expressed RPE markers, as well as phagocytosis, pigments and extracellular matrix‐related markers (Figure S8). Therefore, cluster 12 was identified as the transplanted hESC‐RPE cells. Next, we explored the DEGs between PD‐L1^+^ and CTL hESC‐RPE grafts. Then Gene Ontology (GO) enrichment analysis of the DEGs showed upregulation of survival and repair pathways (e.g., ‘Regulation of development growth’, ‘Response to growth factor’ and ‘DNA damage response’) and downregulation of cell apoptosis and immune response pathways (e.g., ‘Regulation of apoptotic signalling pathway’, ‘Response to reactive oxygen species’, ‘Cytokine‐mediated signalling pathway’ and ‘Interferon‐mediated signal pathway’) in PD‐L1^+^ grafts compared to CTL (Figure 4C). PD‐L1^+^ hESC‐RPE grafts also showed upregulated ‘Tight junction’ pathway, which is important in epithelium function, downregulated immune response and cell apoptosis related pathways, as reflected by KEGG enrichment analysis (Figure 4D). hESC‐RPE cells were further classified into MHC^lo^ and MHC^hi^ hRPE subclusters based on their MHC Class I molecule expression level (Figure 4E,F). PD‐L1^+^ grafts had a greater proportion of MHC^lo^ hRPE, indicating lower immunogenicity than CTL grafts (Figure 4G). Within MHC^lo^ and MHC^hi^ hRPE subsets, immune response and cell apoptosis pathways were also downregulated in PD‐L1^+^ grafts (Figure 4H,I).

### 3.5 PD‐L1 ^+^ hESC‐RPE Grafts Enhance Photoreceptor and Other Retinal Cell Protection in RCS Rats

**FIGURE 5:**
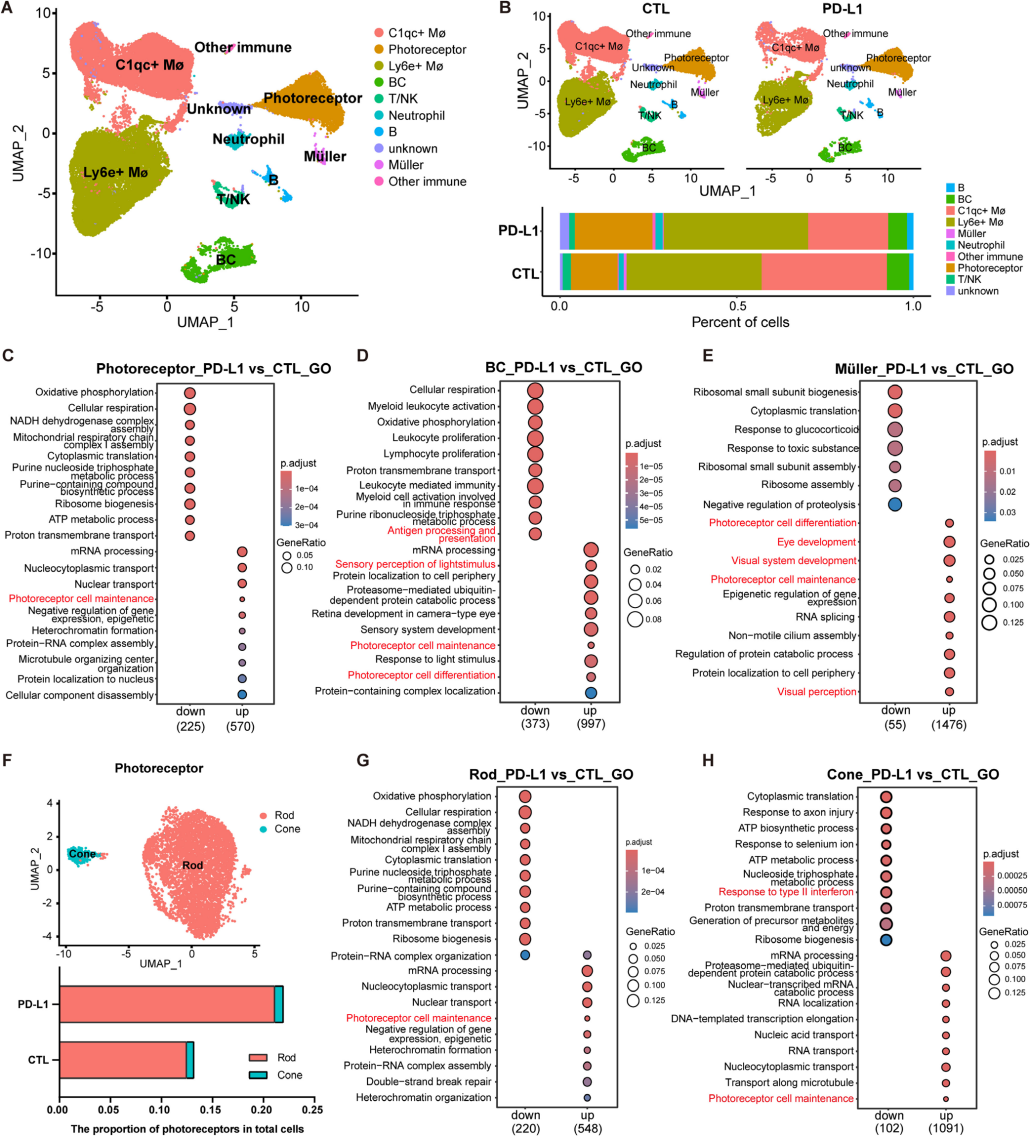
Comparative analysis of PD‐L1^+^ hESC‐RPE‐engrafted rat retinas versus CTL hESC‐RPE‐engrafted rat retinas. (A) UMAP plot of the retinal cells from RCS rats. (B) The CTL and PD‐L1^+^ RPE‐engrafted retina fraction in the indicated cell types. (C–E) GO enrichment analysis of photoreceptors (C), BC (D) and Müller cells (E) in PD‐L1^+^ RPE‐engrafted retinas versus CTL RPE‐engrafted retinas. (F) UMAP plot showing the annotation of RCS rat photoreceptors (upper); the proportion of rod and cone in total rat retinal cells in CTL and PD‐L1^+^ RPE group (lower). (G, H) GO enrichment analysis of rod (g) and cone (h) in PD‐L1^+^ RPE‐engrafted retinas versus CTL RPE‐engrafted retinas.

In order to explore the effect of PD‐L1^+^ hESC‐RPE on the retinal cells of RCS rats, we further extracted and analysed the subpopulations of retinal cells from RCS rats in single‐cell data. We identified different cell populations for rat retinal cells based on gene enrichment within each cluster [42, 43, 44]. A total of 10 clusters were identified: photoreceptor, bipolar cell (BC), Müller cell, C1qc^+^ macrophage/microglia (MØ/MC), Ly6e^+^ MØ/MC, T/NK cell, B cell, neutrophil, other immune cells and unknown cells (Figure 5A). Analysis of cell proportions revealed a higher proportion of photoreceptors, as well as a lower proportion of T/NK cells and C1qc^+^ MØ/MC cells in the PD‐L1^+^ hESC‐RPE group compared to the CTL hESC‐RPE group (Figure 5B). GO enrichment analysis showed that the ‘Photoreceptor cell maintenance’ pathway was upregulated in photoreceptors, BC and Müller cells in the PD‐L1^+^ hESC‐RPE group (Figure 5C–E).

We further subclassified photoreceptors into rod (*Ped6a, Rho, Sag*) and cone (*Arr3, Opn1mw, Gnat2*) clusters (Figure 5F and S9A). Compared with the CTL RPE group, the proportion of rod in the PD‐L1^+^ RPE group was higher, and the proportion of cones was also slightly higher than that in the CTL RPE group (Figure 5F). These results further confirmed that more photoreceptors remained in the retinas from the PD‐L1^+^ RPE group, thereby delaying retinal degeneration. The ‘Photoreceptor cell maintenance’ pathway was also upregulated in rod and cone cluster in the PD‐L1^+^ hESC‐RPE group (Figure 5G,H). Signals related to ROS were downregulated in rod, cone and BC clusters (Figure S9B–D). Moreover, the proportion of cells highly expressing genes involved in the ‘Photoreceptor cell maintenance’ pathway—such as *Mak*, *Cdhr1*, *Pde6a* and *Rho—*was increased in the rod, cone, BC and Müller clusters in PD‐L1^+^ hESC‐RPE engrafted retinas (Figure S9E).

### 3.6 Inhibition of T Cells in the Retina of RCS Rats via the PD‐1/PD‐L1 Axis

**FIGURE 6:**
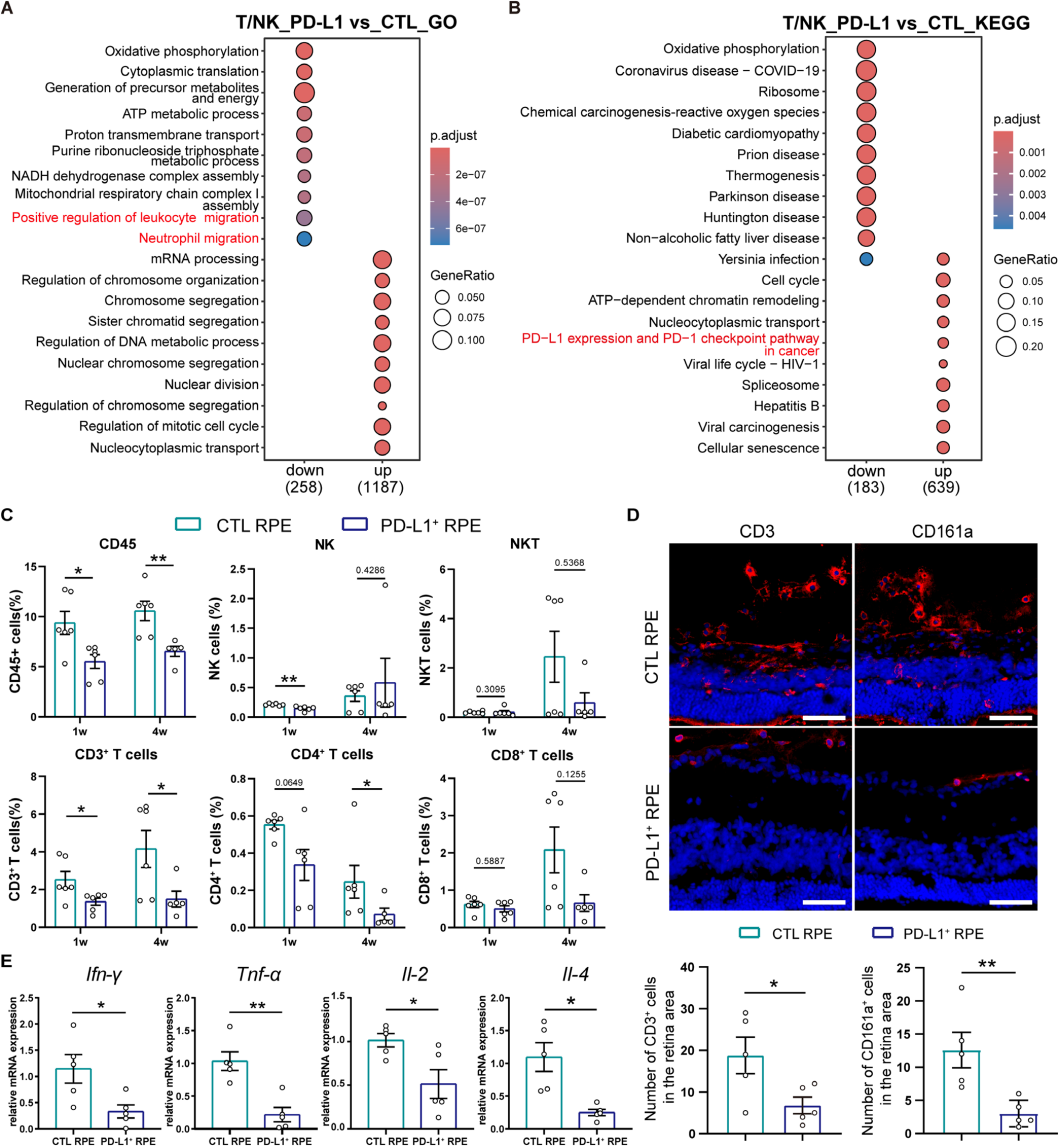
The inhibition of T cell responses via PD‐1/PD‐L1 axis. (A, B) GO and KEGG enrichment analysis of T/NK cells in PD‐L1^+^ RPE‐engrafted retinas versus CTL RPE‐engrafted retinas. (C) Quantification of flow cytometry analysis of immune cells in CTL RPE‐ and PD‐L1^+^ RPE‐engrafted retinas at 1, 4 weeks post‐transplantation. *n* = 5–6 retinas. Mann Whitney test. Data presented as mean ± SEM; *p < 0.05, **p < 0.01. (D) Immunofluorescence images of T cells (CD3) and NK cells (CD161a) in CTL RPE‐ and PD‐L1^+^ RPE‐engrafted eyes at 1 week post‐transplantation (upper). Scale bar, 50 μm. Statistical graph of T cells (CD3^+^) and NK cells (CD161a^+^), *n* = 5 eyes (lower). Unpaired Student's *t*‐test. Data presented as mean ± SEM; *p < 0.05, **p < 0.01. (E) *Ifn‐γ*, *Il2*, *Il4* and *Tnf‐α* expression in CTL and PD‐L1^+^ RPE‐engrafted retinas at 1 week post‐transplantation, as measured by qPCR, *n* = 5 retinas. Welch's *t*‐test. Data presented as mean ± SEM; *p < 0.05, **p < 0.01.

We observed fewer T/NK cells in the PD‐L1^+^ RPE group compared to the CTL RPE group (Figure 5B). To demonstrate T/NK cell inhibition via the PD‐1/PD‐L1 axis, we analysed DEGs in T/NK cells. Pathways such as ‘Positive regulation of leukocyte migration’ and ‘Neutrophil migration’ were downregulated in the PD‐L1^+^ RPE group, while the ‘PD‐L1 expression and PD‐1 checkpoint pathway in cancer’ was upregulated, confirming the inhibitory effects of the PD‐1/PD‐L1 axis (Figure 6A,B).

We then used flow cytometry to measure the proportion of CD45, CD3, CD4, CD8 and CD161 positive cells in the retina of RCS rats 1‐ and 4‐weeks post‐transplantation. Compared to the CTL RPE group, the PD‐L1^+^ RPE group exhibited decreased CD45^+^ immune cells, including CD3^+^ T cells and CD3^−^CD161^+^ NK cells at 1‐week post‐transplantation. This suppression of CD45^+^, CD3^+^ and CD4^+^ cells persisted until 4 weeks post‐transplantation in the PD‐L1^+^ RPE group (Figure 6C). The PD‐L1^+^ RPE group had fewer infiltrating T cells and NK cells in the retina and vitreous (Figure 6D). Additionally, the expression of T cell‐associated cytokines *Ifn‐γ*, *Il2*, *Il4* and *Tnf‐α* was reduced in the PD‐L1^+^ RPE group (Figure 6E). These results confirmed the immunosuppressive effect of PD‐L1 on T cells in the local retinal milieu.

## 4 Discussion

To avoid immune rejection, systemic immunosuppression has been required in all clinical studies to date for allogeneic RPE transplantation. The long‐term influences of systemic immunosuppression on patients are essential for these treatments [45, 46]. Several teams have worked to immune engineer hPSC and its derivatives to escape immune rejection [18]. Sandra et al. developed hESC‐RPE lacking MHC‐I and ‐II, but this strategy led to NK cell activation [47]. Although the authors reported that these NK cells were not cytotoxic, evading NK cell attack in the inflamed retina remains challenging [48]. Both hESC‐RPE and iPSC‐RPE are naturally hypoimmunogenic, expressing HLA I but not HLA II unless stimulated by IFN‐γ [41, 49], and they express HLA‐E, CD47, CD46, CD55, CD59 and secretory factor TGF‐β, which protect RPE from immune attack [25, 50]. T cells play an important role in the rejection of RPE transplantation [25]. However, hESC‐RPE cells were insufficient against T cells in vivo, as our result showed that T cells increased rapidly during rejection. In our study, PD‐1 was upregulated in the rejected retina. PD‐1 was expressed in sites of active inflammation. Previously, PD‐1 expression in retinal neurons in experimental autoimmune uveitis mice was reported [51]. PD‐L1, as the ligand of PD‐1, participates in maintaining the ocular immune privilege [52]. Human and murine RPE naturally expressed PD‐L1 and could be upregulated in the presence of IFN‐γ [53, 54]. Nonetheless, our transplanted hESC‐RPE did not show significant PD‐L1 upregulation at the protein level in the inflammatory retinal milieu. Consequently, we prioritised the PD‐1/PD‐L1 axis, given its prominent role in inhibiting activated T cells [26]. PD‐L1 overexpression as a stem cell protection strategy makes hESC‐RPE grafts approach normal physiological RPE.

In the condition of inflammation in vitro, PD‐L1 promoted the survival of hESC‐RPE by inhibiting T cell proliferation and activation. Due to the immunosuppressive phenotype, hESC‐RPE induced T cell responses in the presence of CD3/CD28 antibody and IL2. Interestingly, CD8^+^ T cells showed a stronger response than CD4^+^ T cells, possibly due to the lack of HLA‐II expression in hESC‐RPE, which primarily activates CD4^+^ T cells [55]. In our results, PD‐L1 mostly suppressed CD8^+^ T cells in vitro. On the contrary, PD‐L1^+^ hESC‐RPE inhibited CD4^+^ T cells more than CD8^+^ T cells in rat retinas and even inhibited NK cells. We suspect that CD4^+^ T cells and NK cells in the rodent retinas express PD‐1 [56, 57].

PD‐L1^+^ hESC‐RPE grafts exhibited enhanced survival and visual preservation in two rodent models of retinal degeneration (RSC rats and RD10 mice). RD10 mice, which are mainly affected by photoreceptor degeneration rather than RPE damage, are not suitable for research on RPE replacement studies, however, to reduce the contingency of the results caused by a single model of RCS rats, we used RD10 mice as another degeneration model just to verify the survival difference between CTL RPE and PD‐L1^+^ RPE. Although the protection of PD‐L1 on transplanted tissues has been examined before, the states of grafts were not reported. To understand the in vivo changes and the protective effects on host retinal cells of PD‐L1^+^ hESC‐RPE, we performed scRNA‐seq of RCS rat retinas that received CTL and PD‐L1^+^ RPE transplantation. While all retinal cells were isolated for scRNA‐seq, our goal was to capture hESC‐RPE grafts which later proved challenging. Sorted hESC‐RPE grafts were mixed with rat retinal cells for scRNA‐seq analysis, and a single‐cell map of retinal cells was established. PD‐L1^+^ hESC‐RPE grafts comprised only 0.84% of the total retinal cells, possibly due to sorting‐induced activity reduction. GO and KEGG analysis showed greater survival and lower immune reactivity in PD‐L1^+^ hESC‐RPE grafts, consistent with immunofluorescence findings. Although PD‐L1 mainly suppressed T cells, both adaptive and innate immune response pathways were downregulated in PD‐L1^+^ hESC‐RPE grafts, decreasing immunogenicity. PD‐L1^+^ hESC‐RPE grafts contained more MHC^lo^ RPE cells than CTL RPE. We speculated that the improved retinal inflammatory milieu induced by PD‐L1 reduced the stimulating upregulation of HLA molecule expression, and promoted the immune tolerance of RPE grafts. This reduction in inflammation led to lower apoptosis and oxidative stress in the PD‐L1^+^ hESC‐RPE graft [58], with the upregulation of the ‘Tight junction’ pathway, reflecting epithelial protection. Typically, inflammation causes RPE apoptosis, loss of epithelial properties and mesenchymal transformation [58, 59, 60]. However, no functional gene expression differences were observed between PDL1^+^ and CTL cells. For host retinal cells, more photoreceptors were preserved in retinas with PD‐L1^+^ hESC‐RPE‐transplants, companied by enhanced light‐sensing and light transduction signals in photoreceptors (rod and cone), BC and Müller cells compared to CTL RPE, supporting the visual protection provided by PD‐L1^+^ hESC‐RPE. Additionally, oxidative phosphorylation was significantly downregulated in PD‐L1^+^ hESC‐RPE grafts, host photoreceptors and BC, potentially reducing ROS accumulation and cell apoptosis [61].

In line with previous studies, the main immune cells in the retina were microglia/macrophages [62]. C1qc^+^ microglia/macrophage was also reduced in the PD‐L1^+^ hESC‐RPE group. C1qc^+^ macrophages, similar to microglia in the central nervous system, interact with T cells to facilitate phagocytosis and antigen presentation [63, 64]. C1qc expression levels are positively correlated with M1/M2 macrophages and CD8^+^ T cells [65]. In the T/NK cell cluster, the PD‐1/PD‐L1 immune checkpoint pathway was significantly upregulated, while the immune response pathway was downregulated. This indicates that PD‐L1 did inhibit T/NK cells through the PD‐1/PD‐L1 apoptotic signalling pathway. Overall, we believe that the increased survival of PD‐L1^+^ hESC‐RPE grafts and host photoreceptors resulted from reduced immune rejection.

In summary, our study generated immune‐tolerant mature hESC‐RPE cells by overexpressing PD‐L1. PD‐L1 overexpression did not alter the RPE‐functional gene expression. It protected hESC‐RPE from T‐cell cytotoxicity in vitro and in vivo. Furthermore, it promoted the survival of hESC‐RPE grafts in the inflamed retina milieu, providing better visual protection in retinal degenerative rodents. We consider that overexpressing PD‐L1 on hESC‐RPE cells is a potential immune protection strategy for RPE replacement in wet AMD patients. The risk of long‐term immunosuppression against T cells in the local retinal immune milieu is lower than that by systemic immunosuppression. However, there are also some limitations. On one hand, the possible existence of resident memory T cells induces recurrent rejection [66]. On the other hand, our results could be influenced by the immune system incompatibility between rodents and hESC‐derived RPE. In this condition, although PD‐L1 improved RPE xenograft survival, the duration of survival was shorter than anticipated. Moreover, this study only focused on T cell‐mediated responses, ignoring other immune cell, such as B cell, neutrophil and macrophage/microglia in the severe inflammatory retinal milieu, which could also affect the long‐term survival of RPE grafts. Future studies should explore additional immunosuppressive strategies to prolong hESC‐RPE graft survival.

## Author Contributions

Yong Liu and Hongling Liu: conceptualization, writing – review and editing. Bowen Li, Xue Zhang: methodology, software, data curation, writing – original draft. Hongling Liu and Yajie Fang: resources, software, formal analysis. Min Chen, Yuxiao Zeng, Chunge Ren, Qiyou Li: cell culture, transplantation. Chengang Wang, Yingxue Lv, Jia Lu: experiment. Yong Liu: funding acquisition, supervision.

## Conflicts of Interest

The authors declare no conflicts of interest.

## Supporting information


Data S1.

## Data Availability

The scRNA‐seq data reported in the paper have been deposited in the Genome Sequence Archive (GSA: CRA016565) publicly accessible at http://ngdc.cncb.ac.cn/gsa. The scRNA‐seq data of transplanted RPE cells in rabbit subretinal spaces were downloaded from the GEO database with the accession number GSE212896. Further information and requests for resources and reagents should be directed to and will be fulfilled by the lead contact, Yong Liu (liuyong@tmmu.edu.cn).
