# Supplementary material for: PD‐L1 Promotes Immunological Tolerance and Enhances Visual Protection of hESC‐RPE Grafts in Retinal Degeneration
Source: Cell Prolif. 2025 Feb 14;58(8):e70007. doi: 10.1111/cpr.70007 (PMC12336460; doi:10.1111/cpr.70007)
Supplement: Supplementary file 1 — Data S1. [file CPR-58-e70007-s001.pdf]

Supplementary materials

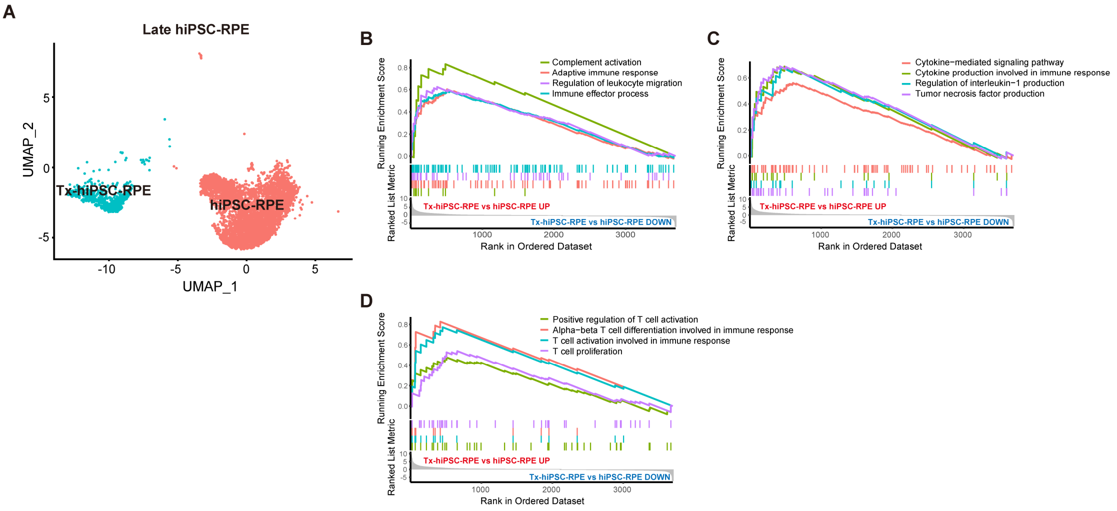

Figure S1. Transcriptomic profiling of hiPSC-RPE cells pre- and post-transplantation

(A) UMAP plot of late (in red) vs. transplanted (in green) hiPSC-RPE cells. Tx-hiPSC-RPE, the transplanted hiPSC-RPE.

(B-D) The GSEA analysis of the immune-related (B), cytokine-related (C) and T cell-related pathways (D) for Tx-hiPSC-RPE vs. late hiPSC-RPE. The single-cell transcriptomic data was downloaded from the public database (GEO database: GSE212896).

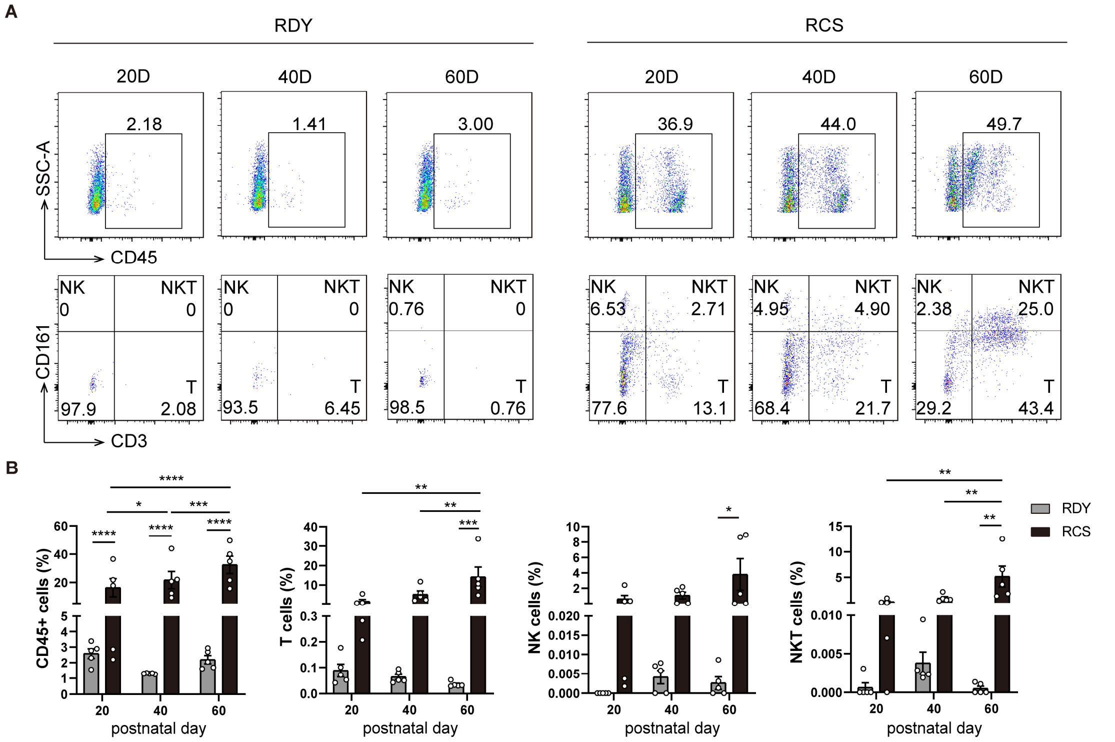

Figure S2. The immunological characteristics of the retina in RCS rats

(A) The representative images of flow cytometry analysis of immune cells ($CD45^+$) in the retina of RCS rats on postnatal day 20, 40, and 60. $CD45^+$ cells were subcategorized as T cells ($CD3^+$), NK

cells (CD3$^-$CD161$^+$) and NKT cells (CD3$^+$CD161$^+$).

(B) The quantification of immune cells in the retina of RCS rats on postnatal day 20, 40, and 60, n=5 retinas/group. Two-way ANOVA. Data presented as Mean ± SEM. *p<0.05, **p<0.01, ***p<0.001, ****p<0.0001.

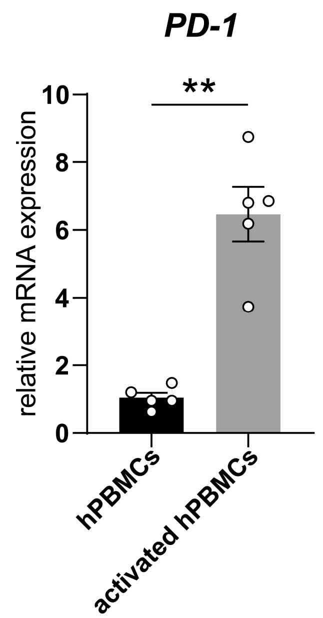

Figure S3. The expression of *PD-1* in hPBMCs

The qPCR analysis of *PD-1* gene expression in unactivated hPBMCs and activated hPBMCs (CD3/CD28), n=5. Welch's *t*-test. Data presented as mean ± SEM; **p<0.01.

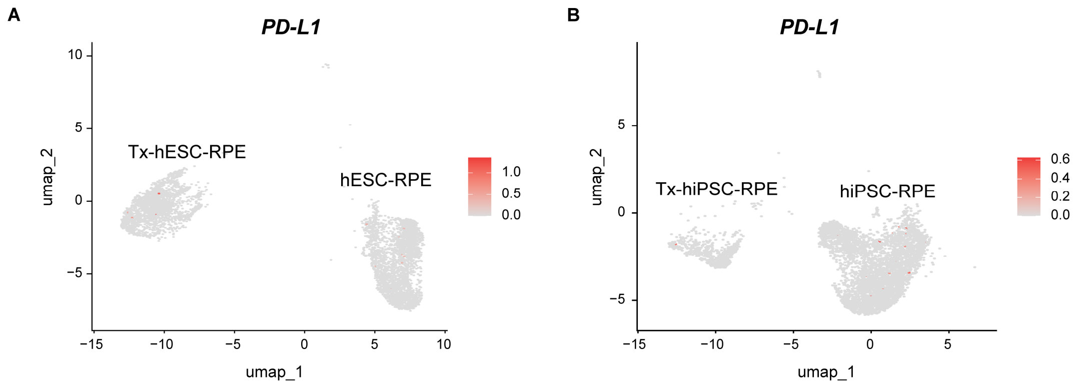

Figure S4. *PD-L1* expression in hESC-RPE and hiPSC-RPE cells pre- and post-transplantation into rabbits

(A) The feather plot shows the expression of *PD-L1* in the hESC-RPE. Tx-hESC-RPE, the transplanted hESC-RPE; hESC-RPE, hESC-RPE cultured *in vitro*.

(B) The feather plot shows the expression of *PD-L1* in the hiPSC-RPE. Tx-hiPSC-RPE, the transplanted hiPSC-RPE; hiPSC-RPE, hiPSC-RPE cultured *in vitro*.

The single-cell transcriptomic data was downloaded from the public database (GEO database: GSE212896).

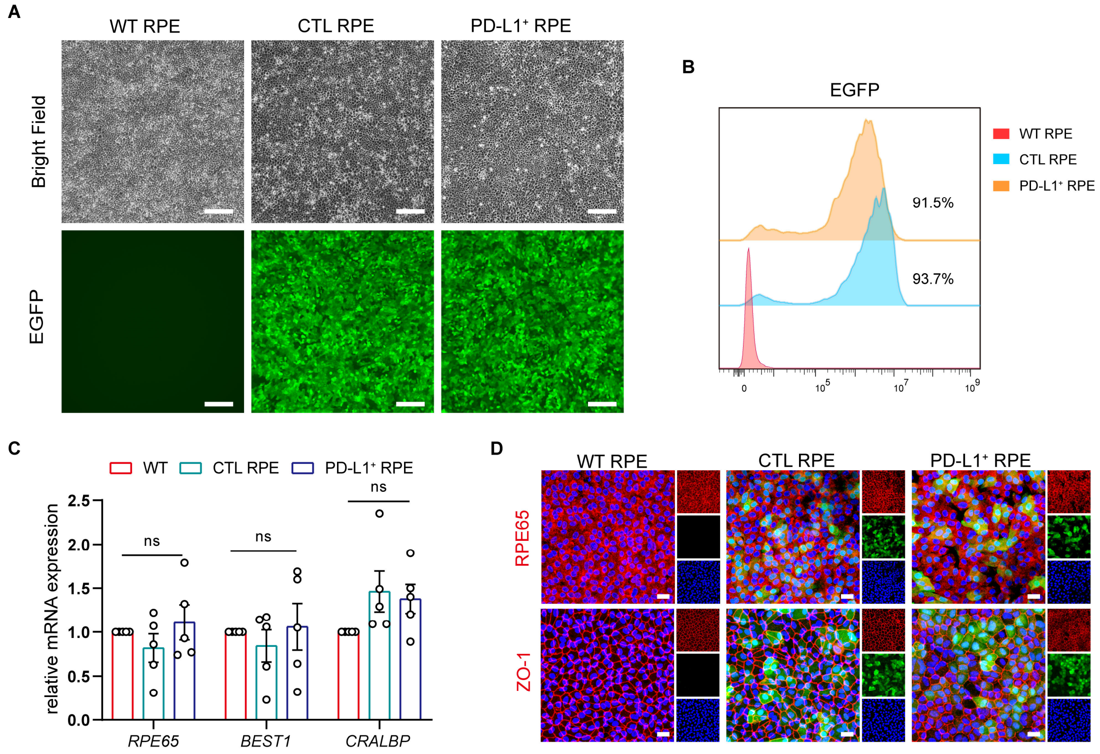

Figure S5. The identification of PD-L1^{+} hESC-RPE

(A) The morphology of hESC-RPE cells under white light (BF) and fluorescence (EGFP). WT RPE, hESC-RPE cells without lentiviral transduction; CTL RPE, hESC-RPE cells transduced with lentivirus carrying empty vectors; PD-L1^{+} RPE, hESC-RPE cells transduced with PD-L1 overexpression lentivirus. Scale bar, 200 μm.

(B) Flow cytometry analysis of EGFP^{+} cells.

(C) Mature RPE marker gene expression in WT, CTL and PD-L1^{+} hESC-RPE cells, as measured by qPCR, n=5 independent experiments. One-way ANOVA followed by Tukey's post hoc test. Data presented as Mean ± SEM. ns: not significant.

(D) Immunofluorescence images of RPE65 (RPE marker) and ZO-1 (cell tight junction marker) of WT, CTL and PD-L1^{+} hESC-RPE cells. Scale bar, 20 μm.

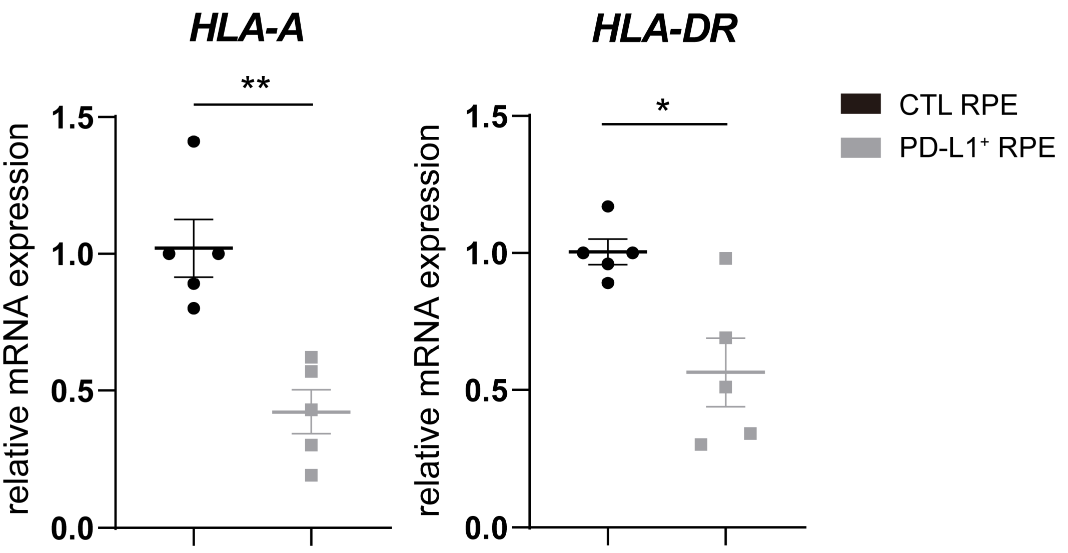

Figure S6. The expression of HLA molecules in CTL and PD-L1$^+$ hESC-RPE after coculture with hPBMCs

The qPCR analysis of HLA-I (*HLA-A*) and HLA-II (*HLA-DR*) gene expression in CTL and PD-L1$^+$ hESC-RPE cells, n=5. Unpaired Student's *t*-test. Data presented as mean ± SEM; *p<0.05, **p<0.01.

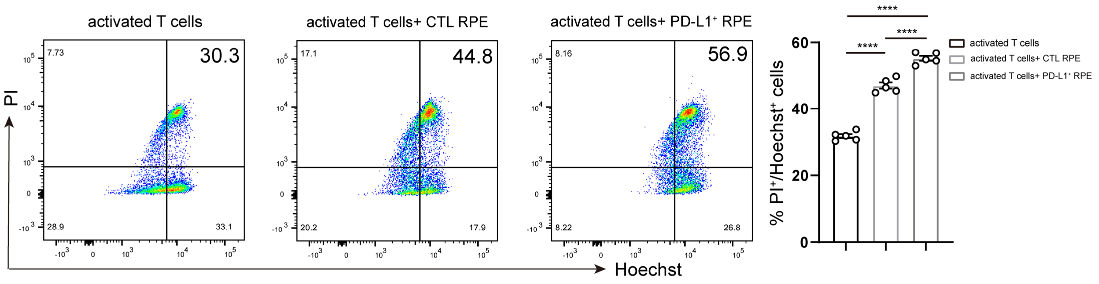

Figure S7. T cell apoptosis after coculture with CTL/PD-L1$^+$ hESC-RPE

Flow cytometry analysis of PI$^+$/Hoechst$^+$ apoptotic T cells after coculture with CTL/PD-L1$^+$ hESC-RPE (left); statistical graph (right), n=5. One-way ANOVA followed by Tukey's post hoc test. Data presented as mean ± SEM; ****p<0.0001.

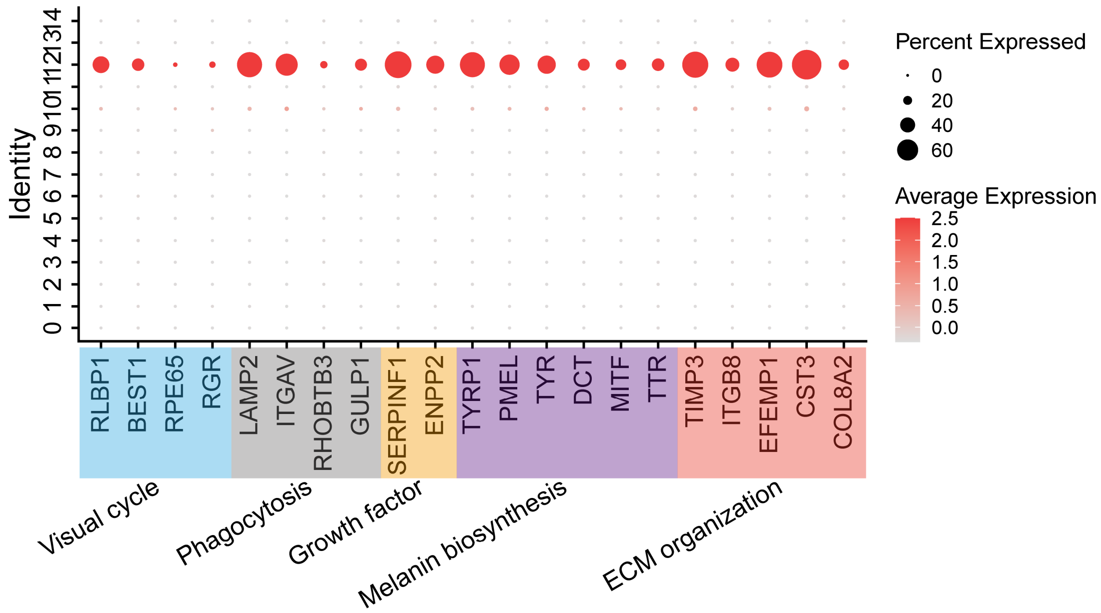

Figure S8. The profile of RPE marker gene expression in transplanted hESC-RPE

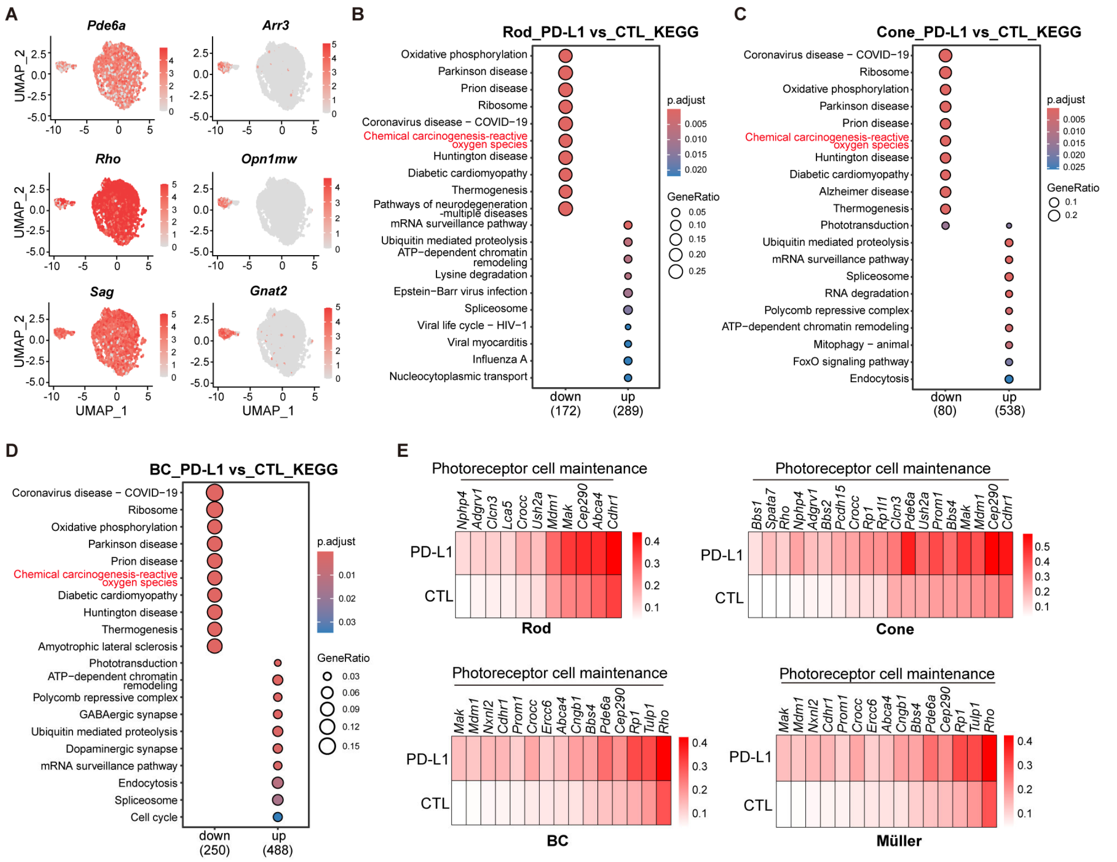

Figure S9. Single-cell transcriptomic analysis of PD-L1⁺ RPE-engrafted retina vs. CTL RPE-engrafted retina

(A) UMAP plots show the expression of marker genes for rod (left) and cone (right).

(B-D) KEGG enrichment analysis of rod (B), cone (C) and BC (D) cells from PD-L1⁺ RPE-

engrafted retinas vs. CTL RPE-engrafted retinas.

(E) Heatmap plots show photoreceptor cell maintenance-associated DEG expression in rod, cone, BC, and Müller cells.

Table S1 primer sequences

Species	Gene	Primer name	Sequence (5'——3')
Human	*PD-L1*	hPD-L1-F	TATGGTGGTGCCGACTACAA
		hPD-L1-R	TGCTTGTCCAGATGACTTC
	PD-1	hPD-1-F	CCAAGGCGCAGATCAAAGAGA
		hPD-1-R	AGGACCCAGACTAGCAGCA
	HLA-A	hHLA-A-F	GCAGTTGAGAGCCTACCTGG
		hHLA-A-R	GGGTCATATGTGTCTTGGGG
	HLA-DR	hHLA-DR-F	ATGGTGTGTCTGAAGCTCCCAG
		hHLA-DR-R	TCCGTCCCATTGAAGAAATG
	RPE65	hRPE65-F	TGCTTACGTACGGGCAATGACTGA
		hRPE65-R	AGTTGGTCTCTGTGCAAGCGTAGT
	BEST1	hBEST1-F	TATGACTCCGGCAGAACACAAGCA
		hBEST1-R	TGTTCATCTCGTTCAGCAGGCTCT
	CRALBP	hCRALBP -F	TTCAAGGGCTTTACCATGCAGCAG
		hCRALBP -R	AGTACCATGGCTGGTGGATGAAGT
	β-ACTIN	hACTIN -F	ACTCTTCCAGCCTTCCTTC
		hACTIN -R	ATCTCCTTCTGCATCCTGTC
Rat	*Tnf-α*	rTnfα-F	CGTCGTAGCAAACCACCAAG
		rTnfα-R	GAGGCTGACTTTCTCCTGGT
	Ifn-γ	rIfn-γ-F	AACAACCCACAGATCCAGCACAA
		rIfn-γ-R	CCAGAATCAGCACCGACTCCTTT
	Il2	rIl2-F	GCTTTCACTTGGAAGACGCTGGAA
		rIl2-R	AAATTCCACCACAGTTGCTGGCTC
	Il4	rIl4-F	TCCTTACGGCAACAAGGAAC
		rIl4-R	GTGAGTTCAGACCGCTGACA
	Pd-1	rPd-1-F	GCGTCTGTGGGTTCTGTGTCG
		rPd-1-R	CCAAGGGTGACTTTAGGTGCTG
	Vista	rVista-F	ACATGGGCTGGAGATAGCTTATG
		rVista-R	TAGATGCTGAGCCTTTGCCTGTC
	Lag3	rLag3-F	GCTCCTGTCCATCTTCCCTG
		rLag3-R	GAAGCGGGTTGGTCACTGTC
	Tim3	rTim3-F	CAGTGTGGTGCTCAGAACGGATG
		rTim3-R	GTAGGTCCCAGAGTCAGCTAGAGTC
	Pd-l1	rPdl1-F	CAGCTTTTGAAGGGGAACGC
		rPdl1-R	TTTGCGGTATGGAGCGTTGA
	Vsig3	rVsig3-F	GCTGCTCGGTGTGGCA
		rVsig3-F	GAGGTTCAGGAGAGCAGCAC
	Gapdh	rGapdh-F	GCCCATCACCATCTTCCAGGAG
		rGapdh-R	GAAGGGGCGGAGATGATGAC
